# How Bioactive Compounds from Brazilian Native Flora of Biopesticide Potential Can Guide Circular Bioeconomy and Sustainability in Agrifood Systems

**DOI:** 10.1021/acsomega.5c01464

**Published:** 2025-06-24

**Authors:** Pedro Henrique Thimotheu Chaves, Anna Paula Azevedo de Carvalho, Carlos Adam Conte-Junior

**Affiliations:** † Research Support Group on Nanomaterials, Polymers, and Interaction with Biosystems (BioNano), Department of Biochemistry, Chemistry Institute, 28125 Federal University of Rio de Janeiro (UFRJ), Rio de Janeiro, Rio de Janeiro 21941909, Brazil; ‡ Center for Food Analysis (NAL), Technological Development Support Laboratory (LADETEC), Chemistry Institute, Federal University of Rio de Janeiro (UFRJ), Rio de Janeiro, Rio de Janeiro 21941598, Brazil; § Nanotechnology Network, Carlos Chagas Filho Research Support Foundation of the State of Rio de Janeiro (FAPERJ), Rio de Janeiro, Rio de Janeiro 20020-000, Brazil; ∥ Graduate Program in Chemistry (PGQu), Chemistry Institute, Federal University of Rio de Janeiro, Rio de Janeiro, Rio de Janeiro 21941909, Brazil

## Abstract

Conventional pesticides, extensively used in agriculture, pose environmental and human health concerns due to their toxicity and bioaccumulation. As sustainable alternatives, biopesticides derived from Brazilian native flora and agro-industrial residues have gained prominence for integrated pest management. This narrative review investigates the pesticidal potential of edible and nonedible plant parts and their alignment with circular bioeconomy strategies. We classified by the type of pesticidal effect (e.g., antifungal, insecticidal, herbicidal, acaricidal, and others). The main bioactive classes reported were flavonoids, terpenes, alkaloids, and phenolic compounds, showing selective activity against phytopathogens such as *Fusarium* spp., *Colletotrichum* spp., and *Aceria guerreronis*. Promising technological strategies included ultrasound-assisted extraction using green solvents, nanoemulsions of essential oils (e.g., *Baccharis reticularia*) for repellent activity, and volatile organic compound profiling from endophytic fungi (e.g., *Induratia* spp.) from coffee stems with nematicidal and antifungal effects. *Arundo donax* was identified for its allelopathic potential, although ecological risks must be considered due to its invasive nature. While antifungal activity is the most frequently reported, other pesticidal classes, especially nematicidal, acaricidal, and herbicidal, remain underexplored and require further validation through in vivo and field studies. Furthermore, most studies relied on in vitro assays; future work must address in vivo efficacy, environmental persistence, safety to nontarget organisms, and scalability of bioactive production. These findings support the role of plant-based biopesticides in sustainable agriculture, aligned with green chemistry principles, and the transition toward a circular bioeconomy.

## Graphical abstract

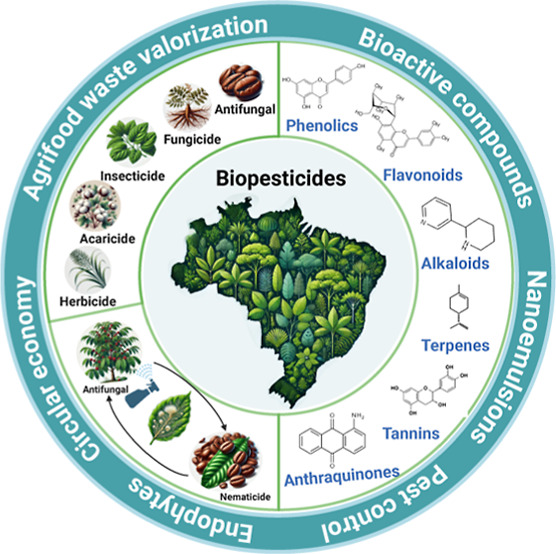

## 1 Introduction

Plant diseases caused by fungi and nematodes represent a major threat to global food security, leading to considerable losses in major calorie crops such as rice, wheat, maize, soybeans, and potatoes.^1^ Fungal infections alone are responsible for 10–23% of annual crop losses, even with the widespread use of antifungal agents.^2^ Nematodes, particularly *Meloidogyne* and *Pratylenchus* spp., also cause severe damage—reducing global yields of crops like soybeans and coffee by up to 10.6% and 15%, respectively.^3–6^ In Brazil, nematodes are estimated to prevent up to 20% of coffee production from reaching the market.^6^ In addition to field losses, postharvest damage caused by stored grain pests such as the red flour beetle (*Tribolium castaneum*) and rice weevil (*Sitophilus oryzae*) compromises food quality and availability.^7^ Furthermore, phytopathogenic fungi are responsible for producing toxic secondary metabolites, such as ochratoxin A from *Aspergillus* spp., which is nephrotoxic, hepatotoxic, and carcinogenic, posing a risk to food safety.^8^ These combined challenges underscore the urgent need for effective and sustainable plant protection strategies.

To address these agricultural challenges, pesticides have become a fundamental technological advancement for enabling large-scale food production. However, multiple studies have reported that certain traditional pesticides may pose risks to human health and the environment.^9–12^ Despite their effectiveness, conventional pesticides—including compounds such as organophosphates, carbofuran, and abamectin—have been associated with significant environmental and human health risks,^13–17^ including carcinogenicity^16^ and neurological disorders.^17,18^ Particularly concerning are the links between cancer and the use of organochlorine pesticides (e.g., dichlorodiphenyltrichloroethane (DDT), dieldrin, and toxaphene) and organophosphates (i.e., phosmet, malathion, and parathion), which have been widely applied in agriculture.^9,10,19^ Moreover, organochlorines are known to bioaccumulate and biomagnify through aquatic ecosystems.^12,20,21^ As a result, regulatory agencies in Brazil, the European Union, and other countries have banned several hazardous formulations, such as Temik (aldicarb) and Gramoxone (paraquat).^22–24^

The dissemination of pesticide-free planting techniques has become a key objective in the developer of greener and safer approaches for sustainable agriculture.^25–27^ This shift is aligned with the principles of green chemistry^28^ and supported by international agendas, including the United Nations call to transform food and agricultural systems to address climate change (UNDP, 2019) and Sustainable Development Goal 2, which aims to “end hunger, achieve food security and improved nutrition, and promote sustainable agriculture”.^29^

In this context, the integration of native plant species and nonedible plant residues into pest control strategies exemplifies a sustainable model aligned with circular economy and green chemistry principles. Figure 1 illustrates this conceptual framework, from the planting of native species to the technological valorization and application of bioactive compounds for phytopathogen control.

**Figure 1:**
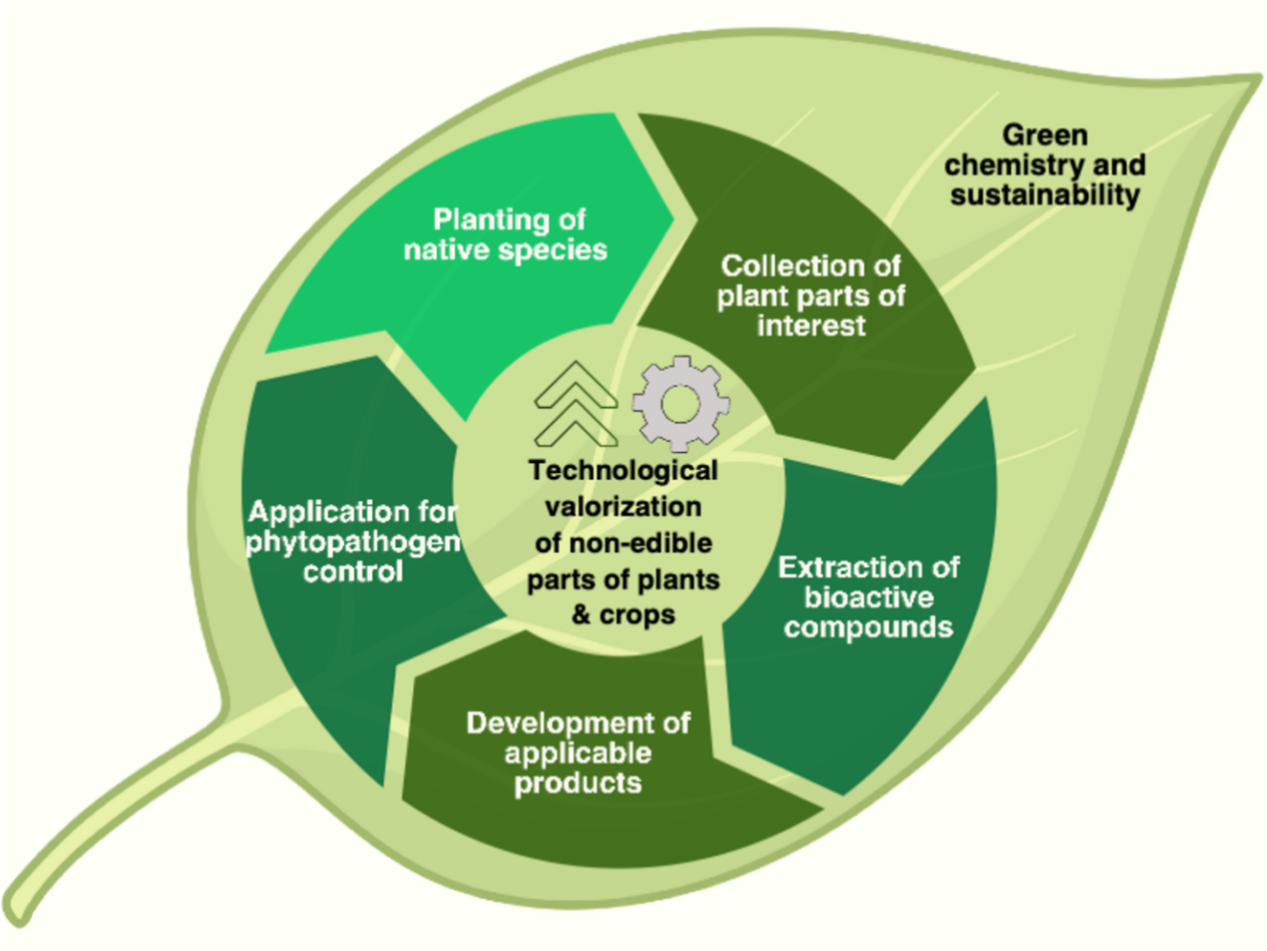
Conceptual framework of sustainable biopesticide development. The green chemistry–aligned valorization cycle of nonedible parts of native plants, including steps from planting to application against phytopathogens.

These concerns have prompted growing interest in alternative pest control strategies. Among them, biopesticides—derived from natural sources such as plants, microbes, or agro-industrial residues—have emerged as promising substitutes for conventional pesticides.^30,31^ Compared to synthetic chemicals, biopesticides offer advantages such as selective toxicity to target pests, biodegradability, reduced environmental persistence, and lower risk to nontarget organisms, including pollinators and humans. Although some formulations may be costly, the use of agricultural residues as raw materials can reduce production costs in certain cases.^32^ Furthermore, exploring native forests and agro-industrial waste for novel biopesticidal compounds provides a valuable pathway toward transitioning from a linear to a circular economy while contributing to ecosystem preservation.^33,34^

Brazil is one of the world’s leading producers of agricultural commodities, including soybeans, sugarcane, coffee, cotton, and stored grains.^35^ The country harbors more than 40,000 native plant species, approximately 20% of the world’s flora, many of which are rich in bioactive compounds.^36^ This remarkable biodiversity positions Brazil as a strategic player in the advancement of green chemistry and the valorization of natural resources.

Phytochemicals are secondary plant metabolites involved in various physiological and ecological functions, including signaling, reproduction, chemical defense, and metabolic regulation.^37^ These compounds, such as phenolics, flavonoids, tannins, alkaloids, and terpenes, exhibit diverse biological activities, including antimicrobial, antioxidant, insecticidal, and allelopathic effects, often through interactions with cellular targets and metabolic pathways.^37^ Their multifunctionality makes them particularly promising for the development of sustainable agricultural inputs.^32^ Figure 2 summarizes representative studies and strategies discussed in this review that illustrate how circular bioeconomy principles guide the extraction and application of pesticidal compounds from Brazilian native flora.

**Figure 2:**
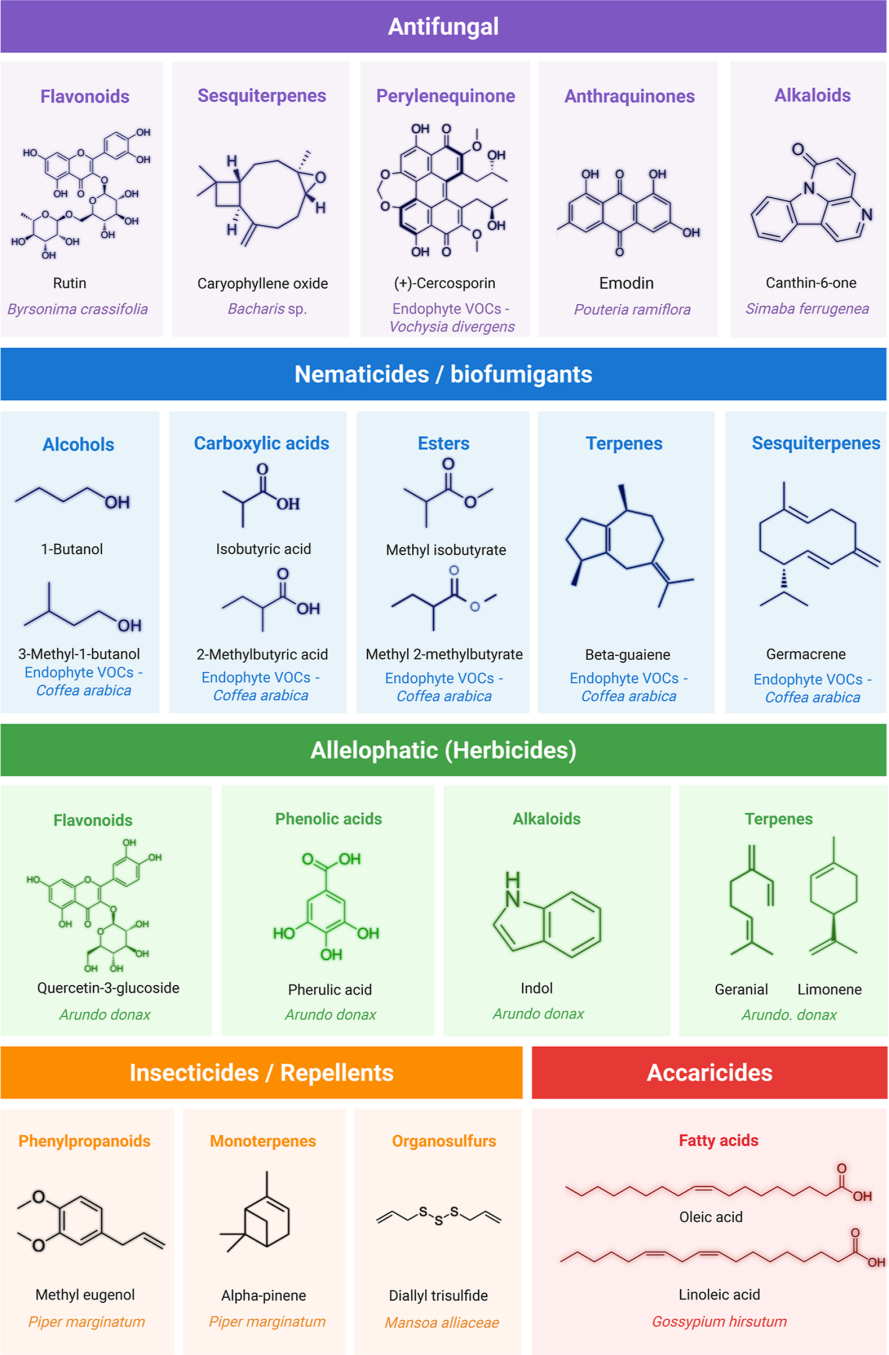
Examples of pesticidal activities (allelopathic, fungicide, nematicide, herbicide, acaricide, repellent, and insecticide) associated with bioactive compounds found in Brazilian native species.

Over the past decade, our research group has focused on the bioprospecting of native Brazilian species from multiple biomes, identifying phytochemicals with antimicrobial and antiparasitic potential.^36,38–42^ Additionally, several plant species from the Caatinga,^43^ Pantanal, and Cerrado biomes have been reported as sources of botanical insecticides, particularly those rich in flavonoids and fatty acids.^43^ Given that flavonoids exert antimicrobial action through multiple mechanisms,^44^ their use as lead compounds in the development of biopesticides against phytopathogens is an emerging and promising strategy.

The detailed literature search strategy adopted for this review is provided in the Supporting Information (Table S1 and Figure S1).

## 2 Main Findings: Bioactive Compounds of Pesticidal Activity

Several studies have employed techniques such as hydrodistillation and steam-distillation^45,46^ to extract bioactive compounds from *Baccharis* sp. However, depending on the phytochemical targeted, heating may cause degradation and reduce yield, particularly for thermosensitive compounds. In such cases, nonthermal techniques like ultrasound-assisted extraction (UAE) have been successfully used to recover phenolic and flavonoid compounds without compromising their integrity.^38,47^

The UAE ethanolic extract of *Byrsonima crassifolia* bark (commonly known as “canjiqueira”) exhibited significant antifungal activity, attributed by the authors to the presence of phenolics, flavonoids, and carboxylic anthraquinones.^48^ These compounds, due to their moderate to high polarity and the presence of hydroxyl and/or carboxyl groups, are highly soluble in polar solvents such as ethanol. UAE enhances the extraction efficiency through cavitation and avoids thermal degradation, preserving compound integrity. Ethanol, being nontoxic and easily removed, further contributes to the sustainability of the process.

Similarly, Girotto et al.^47^ used aqueous and methanolic UAE to extract alkaloids with allelopathic potential from the rhizomes and leaves of *Arundo donax* L. (Poaceae), also known as “cana-do-reino”. Water and methanol proved to be effective for extracting oxygenated and glycosylated compounds such as alkaloids, phenolics, flavonoids, and terpenes. Methanol was particularly effective for extracting oxygenated terpenes, triterpenes, and other functionalized polar compounds. Although toxic and requiring complete removal before biological or food applications, methanol’s high extraction efficiency and ease of elimination have increased its acceptance in green chemistry applications. Figure 3 illustrates a schematic overview of circular bioeconomy strategies used to recover pesticidal bioactive compounds from Brazilian native flora.

**Figure 3:**
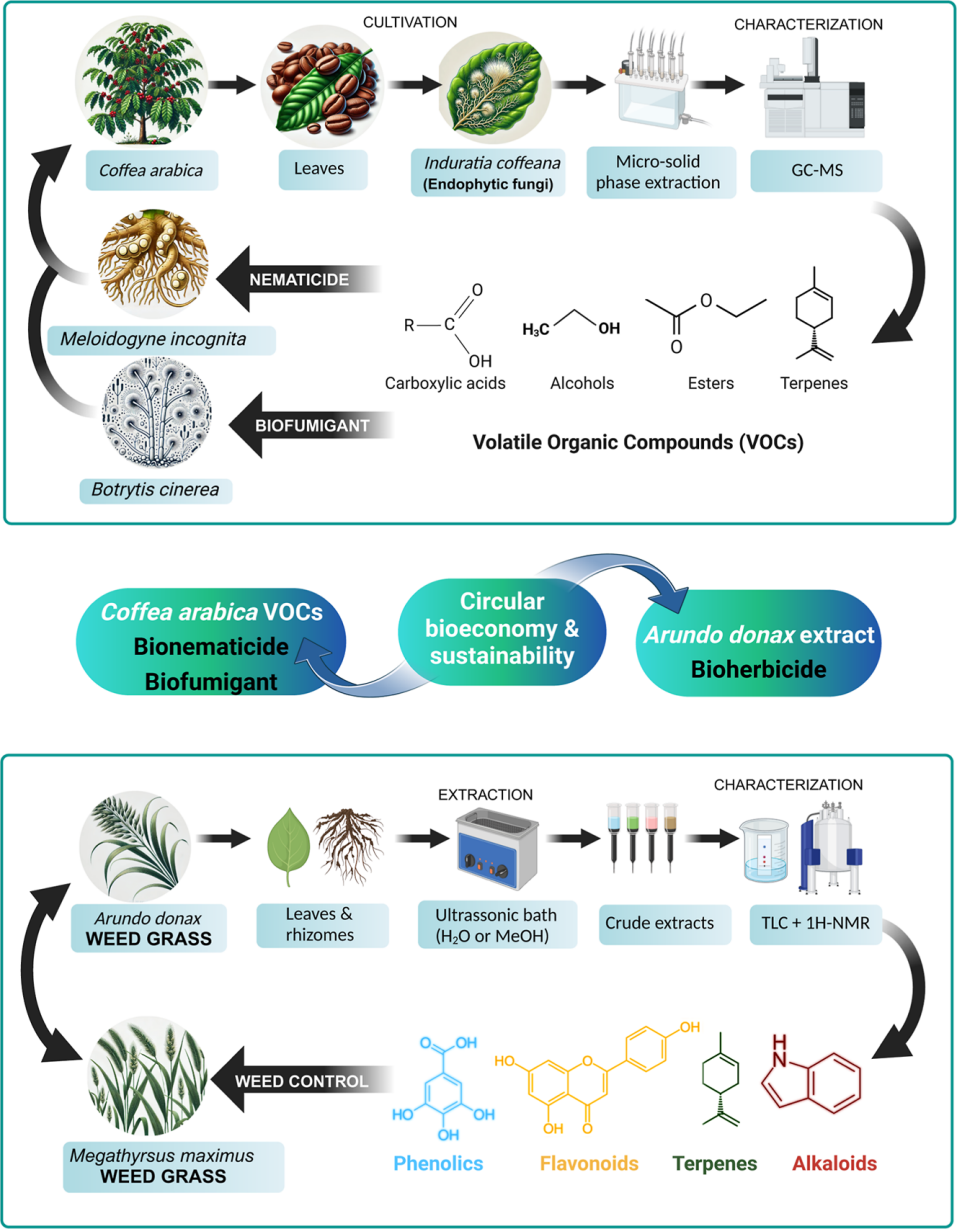
Selected studies reporting nematicidal and fumigant activity (top), and herbicidal activity (bottom) of bioactive compounds extracted from native Brazilian plant species.

Table 1 provides a comparative overview of conventional extraction methods applied to plant-derived biopesticides, analyzing parameters such as yield, energy consumption, solvent use, extraction time, selectivity, operational cost, and compound preservation. Techniques such as maceration,^49,50^ percolation,^8,48,51,52^ and cold steeping^53^ are energy-efficient and suitable for thermolabile compounds, though they typically result in lower yields and limited selectivity. Decoction^54^ and hydrodistillation,^45,46,55,56^ while common, involve thermal exposure that may degrade volatiles or phenolics. UAE, in contrast, offers higher efficiency, reduced processing time, and better preservation of heat-sensitive phytochemicals.

**Table 1:** Comparative Assessment of Extraction Methods Applied to Plant-Derived Biopesticide Compounds

aspect	extraction method					
	cold steeping infusion	maceration	percolation	decoction	hydrodistillation or steam distillation	ultrasound-assisted extraction (UAE)
yield	low to moderate, limited to highly soluble compounds	low to moderate, slow process, depends on time and particle size	low to moderate, efficient for soluble compounds, but dependent on flow rate and setup optimization	moderate, good for polar compounds; volatile may be loss	lower, thermal degradation of sensitive compounds is common	higher, especially for heat-sensitive compounds
energy consumption	very low, no heat or mechanical energy required	low	low	moderate to high, requires constant boiling	high, requires prolonged heating	lower, nonthermal, shorter time
solvent use	water	water or aqueous/nonaqueous solvents	water or aqueous/nonaqueous solvents	water	water or hydrosolvents only	green solvents (e.g., ethanol and water)
time	long	long	long	moderate	long	short
temperature	room temperature	room temperature	room temperature, occasionally under heat	under heat	under heat	room temperature/under heat
amount of organic solvent consumed	high	high	high	none	none	moderate, depends on method and scale
selectivity	low, broad, solvent-dependent extraction	low	low to moderate, depends mostly on solvent polarity	low, broad extraction of water-soluble compounds	low	moderate to high, depending on ultrasound parameters and solvent polarity
operational cost	low, minimal equipment and materials	lower equipment cost and lower energy	lower equipment cost and lower energy	low equipment cost and water only	lower equipment cost, but higher energy	higher equipment cost, but lower energy and time
compound preservation	high, ideal for heat-sensitive compounds	good preservation of thermolabile compounds	good preservation of thermolabile compounds	may degrade heat-sensitive compounds	may degrade volatiles or phenolics	better preservation of thermolabile compounds
references	Malafaia et al.^53^	Gazoni et al.;^50^ de Araújo et al.^49^	Pereira et al.;^8^ Oliveira et al.;^58^ Gomes et al.;^52^ Duarte et al.^51^	Cavichi et al.^54^	Marques et al.;^56^ Xavier et al.;^45^ Lima et al.;^46^ Santana et al.^55^	Andrade et al.;^48^ Girotto et al.^47^

Notably, all reviewed studies employing UAE^47,48^ used ultrasonic bath systems—a milder UAE variant with lower energy and cavitation intensity. No studies reported the use of ultrasonic probe systems, which deliver more intense and uniform cavitation and are considered more efficient, selective, and reproducible.^57^ This methodological gap presents a promising direction for optimizing plant-based biopesticide extraction.

A circular bioeconomy perspective was also evident in the use of endophytic fungi isolated from coffee byproducts (*Coffea arabica*) to combat pathogens affecting the same crop. *Induratia* species were identified as producers of volatile organic compounds (VOCs) with antimicrobial and nematicidal activity, including against *Botrytis cinerea* and *Meloidogyne incognita*.^59^ Although effective in vitro, the large-scale cultivation of *Induratia* spp. and VOC production remains challenging. Moreover, potential effects on nontarget organisms such as soil microbes and pollinators require further investigation.

Among the reviewed studies, some did not involve conventional solvent-based extraction but instead focused on the bioactive potential of the endophytic fungi. For example, Gomes et al.^60^ investigated the antimicrobial and nematicidal activity of VOCs emitted by *Induratia* spp. using solvent-free headspace solid-phase microextraction coupled with GC–MS. Similarly, Godinho et al.^61^ evaluated antifungal activity through direct in vitro interactions without compound isolation. In contrast, studies by Noriler et al.,^62^ Savi et al.,^63^ and Gurgel et al.^64^ used ethyl acetate extraction following fungal culture to recover nonvolatile metabolites. Together, these approaches highlight the biotechnological relevance of endophytic fungi via both VOC-mediated inhibition and secondary metabolite extraction. Table 2 summarizes these methodologies.

**Table 2:** Extraction and Analysis Approaches Used in Studies with Endophytic Fungi Isolated from Brazilian Native Flora^a^

extraction method	endophytic fungal source	compounds analyzed	solvent	analytical approach	observations	refs
direct interaction (coculture without extraction)	Eremanthus erythropappus	not chemically isolated—interaction effects observed	none	coculture bioassays (antibiosis and mycoparasitism)	evaluates ecological interaction; no compound isolation	Godinho et al.^61^
VOCs (solvent-based extraction)	Coffea arabica	VOCs	none	HS-SPME + GC–MS	combined VOC and extract analysis for antimicrobial potential	da Silva Costa Guimarães et al.^59^
		nonvolatile extracts	ethyl acetate	bioassays + MIC/MBC tests		
VOCs collection (solvent-free)	C. arabica	VOCs	none	HS-SPME + GC–MS	highly selective for volatiles; no degradation by solvents	Gomes et al.^60^
solvent-based extraction post fermentation	Vochysia divergens	nonvolatile secondary metabolites (e.g., terpenes and polyketides)	methanol	solvent extraction + GC–MS or bioassay	requires fermentation; preserves broad metabolite profile	Noriler et al.^62^
	Arrabidaea chica		ethyl acetate			Gurgel et al.^64^

a VOCs: volatile organic compounds; HS-SPME: headspace solid-phase microextraction; GC–MS: gas chromatography–mass spectrometry; MIC: minimum inhibitory concentration; and MBC: minimum bactericidal concentration.

Some antifungal studies attempted to identify or associate the mechanisms of action of specific bioactive compounds. Xie et al.^44^ linked the structure of polyphenols—including flavonoids, flavonols, and flavanones—to antimicrobial effects. Andrade et al.^48^ reported that triterpenes exert antifungal effects via membrane disruption and enzyme inhibition. Flavonoids may bind to microbial enzymes, while flavonoids are known to inhibit hydrolytic enzymes produced by phytopathogenic fungi^48,65^ or act as elicitors of plant chemical defenses.^48,66^

Additionally, methanolic extracts of cyanobacteria such as *Nostoc* sp. CENA 219, isolated from benthic freshwater environments in Brazil, have been reported to produce antifungal glycopeptides (e.g., hassallidins) effective against *Candida albicans* and *Aspergillus flavus*.^67^

Teodoro et al.^68^ evaluated the acaricidal effects of crude cottonseed oil (*Gossypium hirsutum* L.), a byproduct of a major Brazilian crop.^69^ The oil demonstrated activity against *Aceria guerreronis* while sparing its natural predator *Typhlodromus ornatus*, highlighting selectivity.

In another approach, Lima et al.^46^ produced a nanoemulsion of *Baccharis reticularia* essential oil, rich in limonene and α-pinene, which showed repellency against *T. castaneum*, a global pest of stored grains. The technique, involving low-energy and solvent-free encapsulation, improved the solubility of lipophilic compounds and their ability to spread and interact with phytopathogens. Figure 4 depicts the primary strategies reviewed for recovering bioactive compounds from Brazilian native flora with a pesticidal potential.

**Figure 4:**
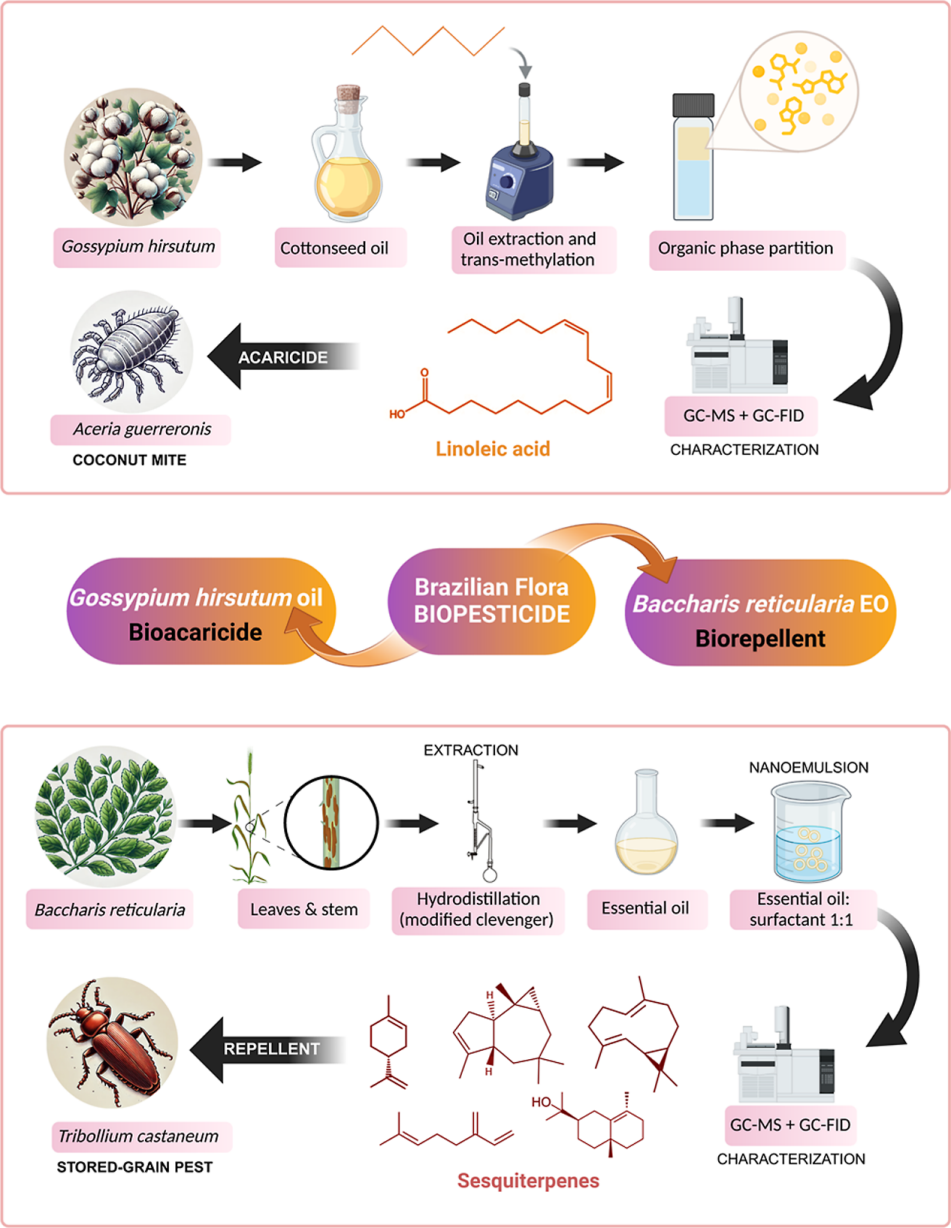
Selected studies reporting nematicidal and antifungal activity (top), and acaricidal activity (bottom) of bioactive compounds extracted from native Brazilian plant species.

### 2.1 Brazilian Biomes as a Source of Plant/Foods Rich in Phytochemicals of Pesticidal Activity

Brazil encompasses six major biomes: Amazon, Atlantic Forest, Cerrado, Caatinga, Pampa, and Pantanal. These biomes represent important natural reservoirs of biodiversity and are considered among the richest sources of phytochemicals with a pesticidal potential. Figure 5 presents the distribution of species collection points by the biome. The Atlantic Forest appears as the most sampled biome, followed by the Pantanal and Amazon.

**Figure 5:**
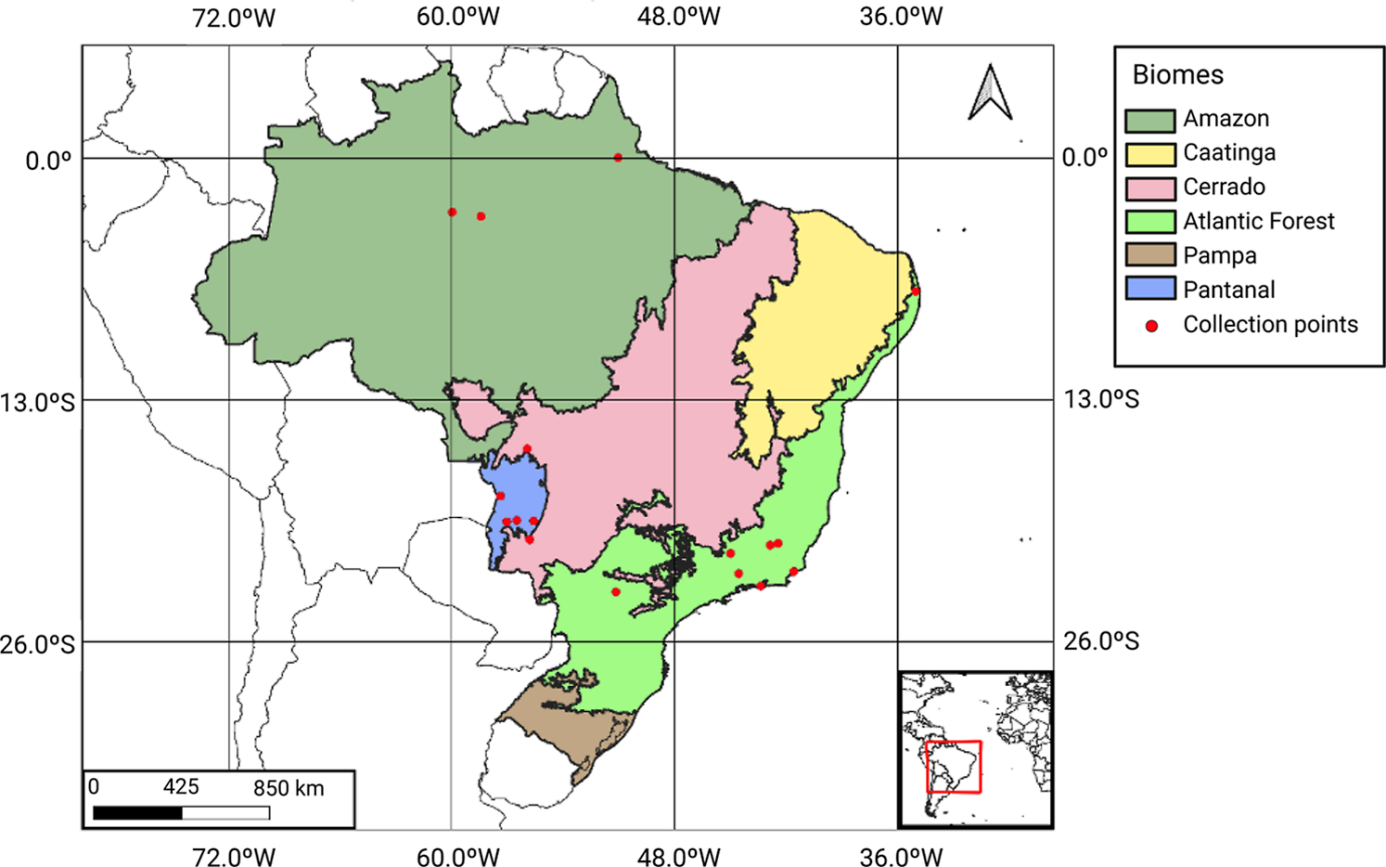
Distribution of species collection points across Brazilian biomes identified as sources of plant species rich in phytochemicls with pesticidal activity. Map generated using QGIS software.

Most studies were concentrated in areas near major urban centers, particularly in the Southeast region. This reflects both logistical accessibility and the scientific infrastructure of these regions. Despite being one of the most degraded and exploited ecosystems in Brazil, the Atlantic Forest still harbors a highly diverse flora, rich in bioactive compounds with biopesticidal properties.

Nevertheless, the Amazon—the largest biome by area and one of the most biodiverse ecosystems on the planet, and the Cerrado biome also deserve greater research attention. The Caatinga (semiarid Northeast) and Pampa (Southern region) are likewise underexplored, even though previous studies have demonstrated their potential as sources of antimicrobial phytochemicals.

### 2.2 Antimicrobial Biopesticides

#### 2.2.1 Phytopathogenic Fungi Causing Plant Diseases

Phytopathogenic fungi are responsible for approximately 80% of plant diseases, significantly affecting crop yields and agricultural productivity.^2,70^ These pathogens are particularly concerning due to their ability to rapidly adapt to fungicides, persist in soil and plant debris, and infect a wide range of economically important crops.^71^ Their impact includes postharvest losses, reduced food quality, and increased production costs, making them a major constraint in both conventional and organic farming systems.^72,73^ The most prevalent soilborne fungal pathogens include *B. cinerea* (gray mold), *Sclerotinia sclerotiorum* (white mold), *Fusarium solani* (fusariosis), *Aspergillus niger* (black mold), *Rhizopus stolonifer* (Rhizopus rot), and *Magnaporthe oryzae*.^7,70^ These fungi were the main biological targets of the biopesticide strategies reviewed in this study. Table 3 summarizes bioactive compounds with antifungal activity derived from Brazilian native flora.

**Table 3:** In Vitro and In Vivo Antimicrobial Effects of Brazilian Native Flora, Classified by Microbial Target Type, with Potential Application in Biopesticide Development for the Control of Fungal and Bacterial Crop Diseases^a^

species	plant part	technology/product form	bioactive compounds	target pathogen (disease)	dose	in vitro outcome	potential use in crop	refs
Antifungal								
Solanum palinacanthum	leaves	rutin isolation (methanolic extraction)	rutin	A. ochraceus (aspergillosis)	568–1.1 μg/mL	MIC: 35 μg/mL (control: 8 μg/mL)	coffee	Pereira et al.^8^
Pectis brevipedunculata	aerial parts	essential oil	terpenoids: geranial, neral, and limonene	A. niger (black mold)	50 mg/mL (1:2)	inhibition zone: 30 mm	grapes and onions	Marques et al.^56^
Pouteria ramiflora	leaves	ethanolic extract (*n*-butanoic fraction)	flavonoids and anthraquinones	Lasiodiplodia theobromae (top root rot)	800–2400 μg/mL	MGRI: 23.9–1.5 mm/day	fruits and vegetable crops	Oliveira et al.^58^
Baccharis dentata and Baccharis uncinella	leaves	essential oil	sesquiterpenes: spathulenol, (*E*)-caryophyllene oxide, δ-cadinene	A. niger (black mold)	4 μL of diluted essential oils (1/5 v/v in ethyl acetate)	qualitative assay: positive growth inhibition	grapes, onions, lettuce, potato, and soy	Xavier et al.^45^
				Rhizopus stolonifer (rhizopus rot)				
				F. solani (fusariosis)				
Byrsonima crassifolia	barks	extracts	phenolics, tannins, flavonoids, anthraquinones, triterpenes, cardiotonic glycosides, and reducing sugars	Fusarium solani (fusariosis)	8–16 μg/mL	5–38% growth inhibition	lettuce, potato, and soy	Andrade et al.^48^
				Sclerotinia sclerotiorum (white mold)	20–24 μg/mL	19–37% growth inhibition		
V. divergens and Stryphnodendron adstringens	leaves and petioles	endophytic fungi extract		Phyllosticta citricarpa (black spot)	mycelial disks (6 mm)	inhibition growth: 90%	citrus and maize	Noriler et al.^62^
				Colletotrichum abscissum (citrus flower rot)		inhibition growth: 70%		
V. divergens	leaves and petioles	endophytic fungi extract	perylenequinones (cercosporin, iso-cercosporin, new polyketide)	P. citricarpa (black spot)	100 μg/disc (control: 1 mg ampicillin)	IZ: 32 mm	citrus and maize	Savi et al.^63^
				C. abscissum (citrus flower rot)		IZ: 50 mm		
		isolated: new phenolic^b^		P. citricarpa (black spot)		IZ: 50 mm		
				C. abscissum (citrus flower rot)		not evaluated		
E. erythropappus	leaves and barks	endophytic fungi isolation		F. solani (fusariosis)	mycelial disks (5 mm)	inhibition: 16%	fruits and vegetables	Godinho et al.^61^
				F. oxysporum (fusariosis)		inhibition: 19%		
				Colletotrichum lindemuthianum (antracnosis)		inhibition: 15%		
				S. sclerotiorum (white mold)		inhibition: 1%		
				Phytophthora sp. (brown rot)		inhibition: 1%		
Maytenus obtusifolia	leaves	ethanolic crude extract	epigallocatechin catechin, epicatechin, phenolics, and tannins	Aspergillus flavus (aspergillosis)	32–1024 μg/mL	MIC: 128 μg/mL	Brazil nut, soy, and peanut	de Araújo et al.^49^
Sideroxylon obtusifolium	leaves	ethyl acetate extract		Thielaviopsis ethacetica (pineapple black rot)	12.5 mg/mL	MG: 0.04 mm/h	pineapple	Duarte et al.^51^
		butanolic extracts				MG: 0.44 mm/h		
A. chica	aerial parts	endophytic fungi extract (ethyl acetate)	terpenes, flavonoids, and phenolic compounds	A. brasiliensis (aspergillosis)	10–0.312 mg/mL	MIC: 2.5 μg/mL	Brazil nut, soy, and peanut	Gurgel et al.^64^
C. arabica	leaves and branches	VOCs of endophytic fungi (Induratia sp.)	sesquiterpenes, alcohols, benzene, and naphthalene derivatives	Aspergillus spp. (aspergillosis)	mycelial disks (5 mm)	growth ratio after 6 days exposure (% vs control): 0 dead	coffee beans	Gomes et al.^60^
Fungicidal								
Simaba ferruginea	rhizomes	alkaloid (0.04% w/w isolated methanolic extract)	canthin-6-one	Aspergillus niger (black mold)	6.25–100 μg/mL	MIC and MFC: 6.25 μg/mL (Amp B: 3.125 μg/mL)	grapes and onions	Gazoni et al.^50^
				Candida spp.		MIC and MFC: 3.125–25 μg/mL (Amp B: 6.25 μg/mL)		
Commelina erecta	stem after flowering	aqueous extract	phenolics (apigenin, luteolin, and quercetin derivatives), organic acids, tocopherols, and sugars	Aspergillus sp.		MIC/MFC: 0.25:0.5 mg/mL	fruit crops	Cavichi et al.^54^
		hydroalcoholic extract				MIC/MFC: 0.5:1 mg/mL		
Antibacterial								
Croton heliotropiifolius	leaves and stem bark	aqueous extract		Ralstonia solanacearum (bacterial wilt)	4.0 mg/mL	inhibition of biofilm formation >50%	tomato	Malafaia et al.^53^
						inhibition of bacterial growth >44%		
A. chica	leaves and branches	endophytic fungi extract (Botryosphaeria mamane)	flavonoids	several sp. of Gram-(+) and Gram-(−) bacteria		MIC: 0.312 mg/mL	fruits and vegetables	Gurgel et al.^64^

a MG: mycelium growth; MIC: minimum inhibition concentration; MFC: minimum fungicide concentration; Amp B: amphotericin B fungicide control; and MGRI: mycelial growth rate index.

b 3-(*sec*-butyl)-6-ethyl-4,5-dihydroxy-2-methoxy-6-methylcyclohex-2-enone; VOCs: volatile organic compounds.

Rutin, isolated from *Solanum palinacanthum* (“joá”) leaves, inhibited *Aspergillus ochraceus* with a minimum inhibitory concentration (MIC) of 35 μg/mL, although this was less potent than the control compound benzalkonium chloride (MIC = 8 μg/mL).^8^ Similarly, essential oil of *Pectis brevipedunculata* (lemongrass), rich in terpenoids such as geranial, neral, and limonene, demonstrated a 30 mm inhibition zone against *A. niger* at 50 mg/mL (1:2 dilution).^56^

An ethanolic extract of *Pouteria ramiflora* (“massaranduba”) wood showed fungicidal activity against *Lasiodiplodia theobromae*, a common citrus pathogen. The *n*-butanol fraction—rich in flavonoids and anthraquinones—was the most effective in reducing the mycelial growth rate index.^58^

The ultrasound-assisted ethanolic extract of *B. crassifolia* (“canjiqueira”) bark inhibited *F. solani* at 8 and 16 μg/mL and *S. sclerotiorum* at 24 μg/mL. The antifungal activity was attributed to phenolic compounds, flavonoids, and carboxylic anthraquinones.^48^ These compounds exhibit moderate to high polarity due to hydroxyl and carboxyl functional groups, making them highly soluble in polar solvents, such as ethanol. The UAE enhanced extraction efficiency through cavitation while preserving thermosensitive structures, and ethanol’s nontoxic, easily removable nature contributed to process sustainability.

A more potent result was observed with canthin-6-one, an alkaloid isolated from *Simaba ferruginea* (“calunga”) rhizomes via methanolic extraction, which showed strong antifungal activity against *A. niger* (MIC = 3.25 μg/mL), comparable to amphotericin B (3.125 μg/mL).^50^ The compound also inhibited *Saccharomyces cerevisiae* and disrupted *Neurospora crassa* cell membranes.

Toxicological evaluations revealed that while the methanolic extract (MESf) exhibited cytotoxicity in CHO-K1 cells (IC_50_ = 16.31 μg/mL), the isolated canthin-6-one was not cytotoxic (IC_50_ = 7.68 μg/mL), and both were classified as nontoxic in vivo at doses up to 1000 mg/kg (MESf) and 100 mg/kg (canthin-6-one), respectively. Despite indications of genotoxic potential in vitro, the overall toxicological risk was comparatively lower than that associated with conventional pesticides.^50^

These findings underscore the potential of sustainable biopesticide formulations to control phytopathogens with efficacy approaching that of synthetic fungicides while offering a reduced toxicological burden.

Complementing these findings, *Maytenus obtusifolia* (“carne-de-anta”) leaves yielded ethanolic extracts rich in flavonols—including epigallocatechin, catechin, and epicatechin—which showed antifungal activity against *A. flavus* with an MIC of 128 μg/mL.^49^

#### 2.2.2 Endophytic Fungi as Biopesticide Sources

Endophytic fungi are nonpathogenic microorganisms that inhabit internal plant tissues and can produce a wide array of bioactive metabolites, including VOCs, with known antimicrobial activity against plant and human pathogens.^74^ Scientific interest in these organisms increased following the discovery of *Induratia alba*, an endophyte isolated from *Cinnamomum zeylanicum*.^75^

Several native Brazilian plants have been identified as hosts of endophytic fungi with antifungal potential, including *Eremanthus erythropappus* (“candeia”),^61^ *Stryphnodendron adstringens* (“barbatimão”),^62^ *Vochysia divergens* (“cambará”),^62,63^ and *C. arabica*.^59^ For instance, *Phaeophleospora vochysiae*, an endophyte from *V. divergens*, produced (+)-cercosporin and (+)-isocercosporin and a novel cyclohexenone derivative. These compounds exhibited inhibition zones of 30–32 mm against *Phyllosticta citricarpa*, while the crude extract also demonstrated moderate activity against *Colletotrichum abscissum* (50 mm), in contrast to the 82 mm inhibition zone produced by the commercial fungicide Derosal (1 mg/disc).^63^

In a subsequent study, *Diaporthe cf. hevea* LGMF 1631—isolated from leaves and petioles of *V. divergens* and *S. adstringens*—inhibited mycelial growth of *P. citricarpa*, *C. abscissum*, and *Fusarium verticillioides* by 75%, 50%, and 50%, respectively, when cultivated in malt extract medium.^62^

Distinct antifungal mechanisms were also reported among endophytes isolated from different plant organs (leaves, bark, and seeds) of *E. erythropappus*. *Diaporthe* spp. inhibited *F. solani* through competitive exclusion; *Trametes villosa* exhibited mycoparasitism; and *Cryptosporiopsis* spp. demonstrated antibiosis, producing inhibition halos.^61^ Quantitatively, *Cryptosporiopsis* spp. inhibited *F. solani*, *Fusarium oxysporum*, and *Colletotrichum lindemuthianum* by 16%, 19%, and 15%, respectively.

Among endophytes with fumigant potential, *Induratia coffeana*—isolated from leaves and stems of *C. arabica*—produced VOCs that qualitatively inhibited *B. cinerea* ^59^ and, more recently, *A. ochraceus* inoculated into coffee beans.^60^ These findings reinforce its potential application as a postharvest biocontrol agent against toxigenic fungi.

This strategy also aligns with circular economy principles by promoting the valorization of coffee byproducts. Coffee is one of Brazil’s most important agricultural commodities, ranking among the top contributors to national GDP.^69,76,77^ However, current practices largely focus on the coffee grain, leaving significant biomass, such as stems and leaves—underutilized. These residues could be repurposed for the development of novel biopesticides, reducing organic waste and increasing added value within the production chain.

In Brazil, the integration of agricultural residues into sustainable bioinput systems is supported by national policies, such as the National Bioinputs Program of the Ministry of Agriculture, Livestock, and Supply,^78^ established by Decree No. 10.375 of May 26, 2020,^78^ and the Bioinputs Law (Law No. 15.070/2024).^79^ These frameworks encourage the transformation of agro-industrial waste into bio-based products with agricultural applications.

Additionally, in vivo toxicological studies have confirmed the safety of extracts from *Sideroxylon obtusifolium* (“quixabeira”) and *Annona acutiflora* (“guiné”), obtained from leaf ethanolic extractions. Both showed no toxic effects on pollinators, supporting their potential for safe in situ application as botanical biopesticides.^51^

#### 2.2.3 Antibacterial Activity of Brazilian Plant Extracts against Phytopathogenic Bacteria

Bacterial phytopathogens can severely impact crop health, and resistance mechanisms, such as biofilm formation, often reduce the efficacy of conventional control methods. In this context, Malafaia et al.^53^ evaluated aqueous extracts from native Caatinga plant species for their activity against *Ralstonia solanacearum*, a soil-borne bacterium responsible for bacterial wilt in several economically important crops.

Although the specific bioactive constituents were not identified, the study focused on antibiofilm effects, an increasingly relevant strategy in bacterial control. Extracts from *Croton heliotropiifolius* (“velame”), *Eugenia brejoensis* (“cutia”), and *Libidibia ferrea* (“pau-ferro”) showed promising results. Notably, the extract of *C. heliotropiifolius* at 4 mg/mL reduced biofilm formation to 8.2% in isolate CGH26 and to 44.5% in isolate B5-5.^53^

In another study, endophytic fungi isolated from *Arrabidaea chica*, a medicinal plant native to the Amazon region, demonstrated antimicrobial activity against both plant- and human-associated bacterial strains. Among the isolates, *Botryosphaeria mamane* CF2-13 showed strong inhibitory effects, particularly against *Staphylococcus aureus* and *Candida parapsilosis*.^64^

### 2.3 Natural Insecticides

The literature reviewed also highlights the potential of biochemical insecticides derived from Brazilian natural products, particularly for their repellency effects, a nonlethal yet effective strategy for pest management (Table 4). One notable example is *B. reticularia* (“alecrim-da-areia”), an endemic aromatic species from the Cerrado and Atlantic Forest biomes, which demonstrated significant repellency activity.

**Table 4:** Pesticidal Activity of Natural Products Derived from Brazilian Native Flora, Categorized by Target Organisms (Insecticidal, Herbicidal, Nematicidal, and Acaricidal Effects)^a^

insecticide								
species	plant part	technology/product form	bioactive compounds	target pest	dose	outcome	potential use in crop	refs.
Repellency Activity								
Baccharis reticularia	leaves and stem	nanoemulsion essential oil	limonene	Tribolium castaneum (red flour beetle)	1.1 μg/cm^2^	**in vitro:** PR-2h: 34% PR-4h: 24%	wheat and corn flour	Lima et al.^46^
			alpha-pinene					
Repellency, Ovicide, and Larvicide Activity								
Piper marginatum	leaves	essential oil	phenylpropanoids: (*E*)-methyl eugenol (34.7%) and (*Z*)-methyl eugenol (27.5%)	Bemisia tabaci (white fly)	^b^LC_50_: 9.39 μL/mL (95% CI)-5 days	**in vitro:***N* = 600, Egg hatch: 11	cotton	Santana et al.^55^
						**greenhouse:** dead nymphs: 18.80; control: 19.4%; efficiency: 44%		
Mansoa alliacea	leaves	essential oil	organosulfides: diallyl trisulfide (52.8%) and diallyl disulfide (33.9%)	Bemisia tabaci (white fly)	^b^LC_50_: 10.99 μL/mL (95% CI)-5 days	**in vitro:***N* = 600, Egg hatch: 14	cotton	Santana et al.^55^
						**greenhouse:** dead nymphs: 9; control: 19.4%; efficiency: 42%		

species	plant part	technology/product form	bioactive compounds	target pest	dose	outcome	crop application	refs
Nematicide								
C arabica	leaves and stems	VOCs of endophytic fungi (Induratia coffeana CML 4011)^c^	terpenes, alcohols, esters, carboxylic acids, and sesquiterpene	Meloidogyne incognita (root-knot nematode)—J2	100 μL of concentrated filtrates	**in vivo:** 24 h: 100% immobility, 48 h: 100% mortality; 6 days: no galls and eggs	root of tomatoes	da Silva Costa Guimarães et al.^59^
Acaricide								
Gossypium hirsutum	seeds	crude cottonseed oil	fatty acids and linoleic acid	Aceria guerreronis (coconut mite) and its predator Typhlodromus ornatus	1 cm disk sprayed with oil	LC_50_: 0.65 μL/mL, more sensible than T. ornatus predator: 5.11 μL/mL	coconut	Teodoro et al.^68^

allelopathic compounds (herbicide potential)								
species	plant part	technology/product form	bioactive compounds	target–invasive species	dose	seed germination	crop application	refs
Arundo donax	leaf	aqueous extract	flavonoid glucosides and terpenes	Handroanthus impetiginosus	10–1.25%	22–57%; control: 90%	lettuce	Girotto et al.^47^
				Eriotheca pubescens		20–60%; control: 77%		
				Guazuma ulmifolia		10–35%; control: 47%		
				Parkia platycephala		10–35%; control: 45%		
				Pseudobombax tomentosum		10–30%; control: 32%		
				Megathyrsus maximus		2.5–45%; control: 45%		

a PR-2h and PR-4h: percentage repellency calculated after 2 and 4 h after the test starts.

b L_50_ (95%CI): lethal concentrations calculated at 95% confidence interval after 5 days of exposure; N: number of individuals.

c Cultivated on J2, during 6–12 days in YES medium, more toxic than potato-dextrose (PAD) medium; J2: second-stage juveniles of *M. incognita*.

Lima et al.^46^ developed nanoemulsions based on EO extracted from *B. reticularia* leaves and stems, as well as its major monoterpene constituents (limonene, α-pinene, and β-pinene), targeting *T. castaneum* (red flour beetle)—a key pest of stored grains. The nanoemulsions were formulated using nonionic surfactants (sorbitan monooleate, polysorbate 80, and/or polysorbate 20). At a concentration of 17.6 μg/cm^2^, both the nanoemulsion and the pure EO achieved 100% repellency after 2 and 4 h of exposure. Remarkably, similar repellency levels (98%) were observed with nanoemulsions at half the concentration (8.8 μg/cm^2^), demonstrating enhanced efficacy.^46^

Moreover, the EO nanoemulsions showed physicochemical stability for up to 90 days under refrigeration and ambient temperature, with no phase separation or significant increase in droplet size.^46^ However, field trials have not yet been conducted, limiting the current assessment of their real-world applicability and environmental persistence. Further research is necessary to validate these findings under field conditions.

In addition, essential oils from Amazonian species such as *Piper marginatum* (“capeba”) and *Mansoa alliaceae* (“cipó-alho”) demonstrated broad-spectrum insecticidal activity against *Bemisia tabaci* MED (silverleaf whitefly), inducing lethal and sublethal effects across all life stages. In both laboratory and semifield greenhouse trials, the EOs exhibited toxicity to nymphs, ovicidal activity, repellency, reduced oviposition, and inhibited colonization. At an LC_50_ of 20 μL/mL, *P. marginatum* and *M. alliaceae* achieved control efficiencies of 91.55% and 90.54%, respectively, with mortality approaching that of the control (18.8 vs 19.4 nymphs).^55^

These effects were attributed to the key bioactive constituents. *P. marginatum* EO contains phenylpropanoids such as (*E*)-methyl eugenol (34.7%) and (*Z*)-methyl eugenol (27.5%), while *M. alliaceae* EO is rich in organosulfur compounds, including diallyl trisulfide (52.8%) and diallyl disulfide (33.9%).^55^

### 2.4 Allelopathic Metabolites as Natural Herbicides

Allelopathic compounds are promising alternatives to synthetic herbicides as they often lack residual toxicity and environmental persistence. These natural phytotoxins can selectively inhibit the germination and growth of weeds, making them suitable for both conventional and organic agriculture. In this context, plant extracts from Brazilian species rich in allelochemicals have been investigated for their herbicidal potential.

*A. donax* L. (Poaceae), an invasive reed found in the Brazilian Cerrado, exhibited significant allelopathic activity in controlled bioassays. Aqueous extracts from its leaves and rhizomes reduced *Lactuca sativa* (*Asteraceae*) seed germination to 39% and 43%, respectively, at 10% concentration. Methanolic extracts were even more effective, lowering germination to approximately 10–12%.^47^ Additional tests with aqueous leaf extracts showed inhibitory effects on five native Cerrado tree species and the invasive grass *Megathyrsus maximus*. The herbicidal activity was associated with the presence of flavonoids, phenolic compounds, and terpenes, as identified by thin-layer chromatography.

The study also highlighted that allelochemicals released from *A. donax* biomass may remain active in the soil, inhibiting the growth of surrounding vegetation—including other invasive species like *M. maximus*.^47^ Given this potential, the authors cautioned against weed control strategies that involve merely cutting *A. donax* to the ground level, as this could unintentionally intensify the release of phytotoxic compounds into the environment and exacerbate its ecological impact.

While the isolation of allelochemicals from *A. donax* may offer herbicidal benefits, the direct use of the whole plant biomass poses environmental risks. As an aggressive invader, further propagating or stimulating the growth of *A. donax* could threaten native plant communities. Therefore, future applications should prioritize the identification, isolation, and formulation of its active compounds rather than relying on the use of intact biomass to avoid reinforcing its invasiveness in biodiversity-sensitive ecosystems.

### 2.5 Bionematicides

In addition to the antifungal and antibacterial effects previously discussed, VOCs and nonvolatile metabolites produced by endophytic fungi of the genus *Induratia* have also demonstrated nematicidal activity. Among all studies reviewed, relatively few focused specifically on nematode control; however, promising results were observed, particularly when using coffee residues, such as roots and stems, as raw materials for developing biopesticides targeting coffee crops.

This approach is especially relevant in Brazil, a leading global coffee producer, where *M. incognita* is a major phytopathogen responsible for significant yield losses and substantial economic damage annually.^51^

One study evaluated VOCs produced by *I. coffeana* CML 4011 grown on *C. arabica* leaves over different time intervals.^59^ After 6 days, nematode mobility was reduced to 14.9%, with no gall formation or egg production. By day nine, mobility reached 13.0%, still with no galls observed, and 54.9 eggs were counted. After 12 days, mobility was 15.4%, with no galls and a reduced egg count of 37.2. These inhibitory effects were attributed to VOCs comprising terpenes, alcohols, esters, carboxylic acids, and sesquiterpenes.

In addition, filtrates from the fungal culture medium of *I. coffeana* showed remarkable nematicidal activity. At 100% (v/v) concentration, the extract induced 100% immobility within 24 h and complete mortality of nematodes within 48 h.

Despite these promising findings, several challenges hinder the practical application of *Induratia*-derived compounds in agricultural settings. The large-scale cultivation of endophytic fungi is constrained by variability in growth behavior, specific culture conditions, and limited yields or volatility of certain bioactive metabolites. Moreover, the feasibility of industrial-scale VOC production remains largely untested.

Furthermore, although these volatiles are effective against plant pathogens and pests, their potential impact on nontarget organisms—such as pollinators, beneficial soil microbiota, and mycorrhizal fungi, is yet to be determined. Therefore, future studies must include comprehensive ecotoxicological assessments to ensure that the field application of fungal VOCs does not compromise soil health, ecosystem function, or biodiversity.

### 2.6 Natural Acaricides

Brazilian native plant species have also been explored for their potential as natural acaricides, pesticides that target mites, including phytophagous species and, in some cases, their natural predators. Mites of agricultural relevance, such as *A. guerreronis*, are major pests in crops like coconut, while predatory mites (e.g., *T. ornatus*) serve as biological control agents in natural and cultivated ecosystems.

Teodoro et al.^51^ evaluated the acaricidal activity of crude cottonseed oil (*G. hirsutum* L.), a byproduct of cotton processing and a crop of significant economic importance in Brazil.^69^ The study demonstrated promising results with high selectivity toward the target pest. The mortality rate for *A. guerreronis* was significantly higher than that observed for its natural predator *T. ornatus*, with LC_99_ values of 1.60 μL/cm^2^ and 50.46 μL/cm^2^, respectively.

These findings suggest that cottonseed oil exhibits both toxic and repellent effects on *A. guerreronis* while maintaining a considerable margin of safety for predatory mites (*Acari*: *Phytoseiidae*). This selectivity represents a key ecological advantage in integrated pest management strategies, particularly in commercial coconut plantations, where conserving predatory mite populations is essential for long-term pest control.

However, despite these benefits, the long-term safety and environmental fate of plant-derived acaricides remain poorly understood. Specifically, data on soil persistence, degradation rates, or bioaccumulation of active compounds (e.g., environmental half-life) are lacking for many botanical biopesticides.^80,81^ Therefore, future studies should assess the environmental stability and potential nontarget impacts of such compounds under field conditions, ensuring that their continued use does not compromise soil health, biodiversity, or ecosystem services.

## 3 Conclusions and Perspectives

This review underscores the broad potential of Brazilian native flora and agro-industrial residues as sources of sustainable biopesticides. Although significant attention has been given to antifungal activity, other categories, such as nematicides, acaricides, herbicides, and insect repellents, remain underexplored. For instance, the antifungal properties of phenolic compounds and flavonoids from *B. crassifolia* and *M. obtusifolia* showed promise in inhibiting phytopathogens like *F. solani* and *Aspergillus* spp., while alkaloids such as canthin-6-one from *S. ferruginea* presented comparable potency to commercial fungicides.

Among insecticidal approaches, nanoemulsions of EO from *B. reticularia* demonstrated enhanced repellency against *T. castaneum*, although field trials are still lacking. VOCs emitted by endophytic fungi like *I. coffeana*, cultivated from coffee residues, exhibited nematicidal and antifungal activity, representing a circular bioeconomy approach; however, scalability and ecotoxicological assessments remain key challenges for real-world application. Likewise, allelopathic compounds from *A. donax* showed herbicidal activity against native and invasive weeds, but their application demands careful environmental risk analysis due to the invasive nature of the source species.

Overall, most biopesticidal effects reviewed were confirmed under in vitro conditions, suggesting that in vivo and field studies are critical next steps. Future research should prioritize: i. standardized toxicity and environmental persistence assays (e.g., soil half-life, impact on pollinators, and beneficial microbes); ii. optimization of extraction technologies (e.g., use of high-power ultrasonic probes in UAE); iii. isolation and formulation of lead bioactives with known modes of action; and iv. pilot-scale validation of compounds sourced from underutilized biomass, especially from high-volume agricultural sectors such as coffee and cotton.

These strategies will be fundamental to advancing the development of biopesticides that are not only effective and selective but also scalable, economically viable, and aligned with circular economy and biodiversity conservation principles.

## Supplementary Material



## Notes

Eligibility criteria for article selection and PDF full-reading step in the review process and flowchart with steps and primary results of the systematic selection and reading (PDF)
