# Supplementary material for: How Bioactive Compounds from Brazilian Native Flora of Biopesticide Potential Can Guide Circular Bioeconomy and Sustainability in Agrifood Systems
Source: ACS Omega. 2025 Jun 24;10(31):33965–81. doi: 10.1021/acsomega.5c01464 (PMC12355236; doi:10.1021/acsomega.5c01464)
Supplement: Supplementary file 1 [file ao5c01464_si_001.pdf]

Supporting Information: How bioactive compounds from Brazilian native flora of biopesticide potential can guide circular bioeconomy and sustainability in agrifood systems

Pedro Henrique Thimotheu Chaves [1,2][1], *Anna Paula Azevedo de Carvalho* [1-4*][2], *Carlos Adam Conte-Junior* [1-4]

[1] Research Support Group on Nanomaterials, Polymers, and Interaction with Biosystems (BioNano), Department of Biochemistry, Chemistry Institute, Federal University of Rio de Janeiro (UFRJ), Rio de Janeiro, RJ 21941909, Brazil.

[2] Center for Food Analysis (NAL), Technological Development Support Laboratory (LADETEC), Chemistry Institute, Federal University of Rio de Janeiro (UFRJ), Rio de Janeiro, RJ 21941598, Brazil.

[3] Nanotechnology Network, Carlos Chagas Filho Research Support Foundation of the State of Rio de Janeiro (FAPERJ), Rio de Janeiro RJ 20020-000, Brazil.

[4] Graduate Program in Chemistry (PGQu), Chemistry Institute, Federal University of Rio de Janeiro, Rio de Janeiro, RJ 21941909, Brazil.

Literature Search methods

To increase the quality and confidence of this narrative review, the authors used several tools and strategies commonly employed in systematic reviews. The main results of each step from evidence recovery are displayed in the Supporting Information by Figure S1. The search was conducted according to a predefined search protocol to investigate the state of the pesticidal effects of edible and non-edible parts of Brazilian native flora, which parts of plants have been studied. Embase, Science Direct, Scopus, and Web of Science databases were chosen as search sources. The initial screening step was performed in March 2022, and an update was made by April 2024. The returned articles' citation files (.ris or .bibtex) were cataloged and stored using a review manager, the StArt 2.3.4.2 tool [41]. The initial selection of articles was conducted by reading the titles, abstracts, and keywords. Records that did not meet eligibility criteria were excluded. In general, the eligibility criteria (Supporting Information, Table S1) were defined to

[1] Pedro Henrique Thimotheu Chaves and Anna Paula Azevedo de Carvalho contributed equally to this manuscript, and each has the right to list themselves first in author order on their CVs.

[2] Corresponding author: Anna Paula Azevedo de Carvalho - Department of Biochemistry, Chemistry Institute, Federal University of Rio de Janeiro, Rio de Janeiro, RJ 21941909, Brazil. ORCID: https://orcid.org/0000-0001-9258-5947 E-mail address: annacarvalho@iq.ufrj.br

answer the main research focus and questions: identify plants from the Brazilian region and biome; which are the main species used to recover metabolites with phytopathogenic activities; Of these, which part of the plant (edible or non-edible) was used? Which bioactive compounds recovered have phytopathogenic activity? Our objective is to classify and discuss the findings of pesticide activity according to the target organism (fungicide, herbicide, insecticide, bactericide, acaricide, and others). Moreover, we also purpose to comprise the primary pesticidal mechanism of action of Brazilian natural products discussed in this review.

A manual search for published articles was carried out using external sources, including a list of references for the included articles. Authors defined keywords and synonyms for terms composing component search SC1) "Pantanal", "Amazônia", "Cerrado", and "Mata Atlântica" to refer to the biome/region of Brazil; SC2) "pesticide", "biopesticide", and "antimicrobial", related to the biological activity; and SC3) "phytochemicals", "bioactive compounds", and "flavonoids" for associated phytochemical compounds. The search components (SC1 + SC2 + SC3) comprise general search strings adapted for each database. For advanced research in databases to retrieve research articles, combined three search components (SC) using Boolean operators "AND" and "OR". The search string is used in all databases.

The eligibility criteria used to select original articles included in the identification and full-text reading step are described in Supporting Information by Table S1. As auxiliary tools, four databases of chemical and biological information from Brazilian biodiversity were used to confirm the common name, species, and most frequent biome/region of the species: "NuBBED" (https://nubbe.iq.unesp.br/portal/nubbe-search.html), "SiBBr" (https://www.sibbr.gov.br/), "Flora Digital" for biodiversity of South region of Brazil (https://floradigital.ufsc.br/), and REFLORA/CNPq (https://floradobrasil.jbrj.gov.br/reflora). After that, the authors used the QGIS software and IBGE shapefiles to obtain a cartographic basis for preparing the biome map with information on the collection point of species found in this study to bring the geographic information of species.

Table S1. Eligibility criteria for article selection and PDF full-reading step in the review process.

**Inclusion criteria**	**Exclusion criteria**
Articles studying a plant species native from Brazil	Comments, reviews, editorials, thesis, conferences, books, abstracts, preprints, hypothetical and modeling studies
Articles studying at least one pesticidal effect (insecticide, fungicide, herbicide, acaricide, etc)	Article reporting the pesticidal effect of Brazilian biodiversity of non-plant origin
Article reporting bioactive compounds found in natural products from Brazilian native flora	Article reporting Brazilian native species without evaluating pesticidal effect or bioactive compounds associated
Articles studying pesticidal mode of action of Brazilian natural products	Articles did not attend the study's purposes or did not mention any native species from Brazil

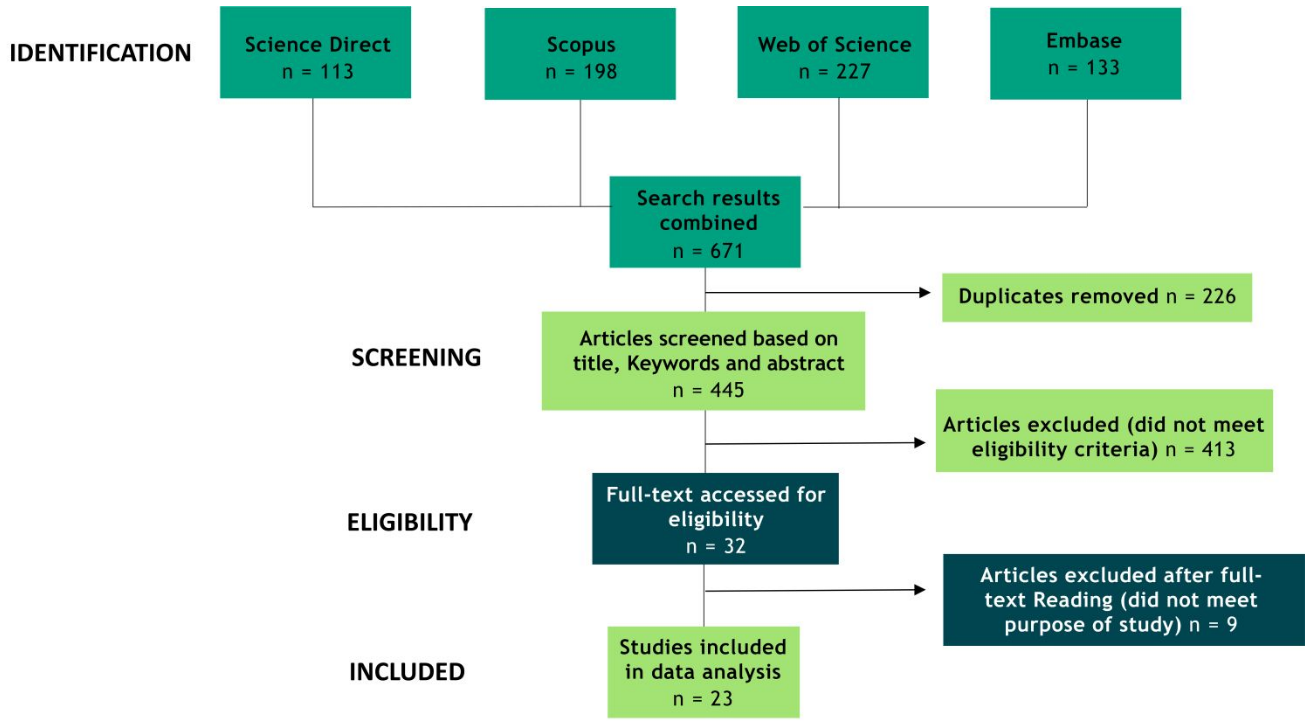

Figure S1. Flow chart of primary results of the systematic selection and reading.
